# Association between the preoperative serum albumin-to-creatinine ratio (sACR) and in-hospital mortality in perioperative patients with chronic kidney disease: a cohort study based on INSPIRE database

**DOI:** 10.1080/0886022X.2026.2621525

**Published:** 2026-02-04

**Authors:** Bin Pan, Bingwen Lin, Xiurong Huang, Xiaochen Zhang

**Affiliations:** a Department of Anesthesiology, Fujian Provincial Governmental Hospital, Fuzhou, China; b Department of Critical Care Medicine, The First Affiliated Hospital of Fujian Medical University, Fuzhou, China

**Keywords:** Chronic kidney disease, albumin, creatinine, serum albumin-to-creatinine ratio, mortality

## Abstract

**Objective:** The serum albumin-to-creatinine ratio (sACR) is a potential biomarker for multiple diseases, yet its prognostic role in chronic kidney disease (CKD) patients undergoing surgery remains unexplored. This study aimed to investigate the association between sACR levels and postoperative in-hospital mortality in this population. **Methods:** This retrospective cohort study analyzed 2,611 CKD patients from the INSPIRE database (2011–2020). Patients were stratified into tertiles based on admission sACR levels. Multivariable Cox regression models were used to estimate hazard ratios (HRs) and 95% confidence intervals (CIs) for the association between sACR and mortality. Restricted cubic spline (RCS) analysis was performed to assess potential nonlinear relationships. Sensitivity analyses were conducted to verify result robustness. **Results:** The cohort had a mean age of 61.1 ± 14.8 years and a median sACR of 1.2 (interquartile range: 0.7–2.6). Multivariable analysis revealed that each unit increase in sACR was associated with a 29% reduction in mortality (adjusted HR 0.71, 95% CI 0.61–0.83, p &lt; 0.001). Compared with the lowest tertile (T1), the highest tertile (T3) demonstrated a 48% lower mortality risk (HR 0.52, 95% CI 0.33–0.82, p = 0.005). RCS analysis identified a nonlinear, L-shaped association between sACR and mortality (P for nonlinearity &lt; 0.001), with a threshold effect observed at approximately 2.7. Sensitivity analyses confirmed the robustness of these findings. Conclusion: Lower sACR levels are independently associated with higher in-hospital mortality in surgical CKD patients, exhibiting an L-shaped relationship with a critical threshold at 2.7. sACR represents a practical, cost-effective early-warning biomarker for perioperative risk stratification in this high-risk population.

## Introduction

Chronic kidney disease (CKD), defined by reduced estimated glomerular filtration rates (eGFRs), structural abnormalities (e.g., irregular morphology on imaging), or persistent urinary markers (albuminuria [ACR ≥30 mg/g] or hematuria [≥5 RBCs/HPF]) lasting >3 months [1,2], has a global prevalence of approximately 10%, affecting an estimated 697 million individuals worldwide [1,3–5]. With an aging population and better survival rates for end-stage kidney disease, surgeries on patients at all stages of CKD are becoming more common. CKD patients undergoing surgical procedures face a markedly higher risk of postoperative complications, including acute kidney injury (AKI), infections, cardiovascular events, and prolonged hospital stays [6]. The perioperative period poses unique challenges for these patients, as the physiological stress of surgery, anesthesia, and potential nephrotoxic interventions can exacerbate underlying renal dysfunction [7–9]. Therefore, identifying reliable biomarkers to predict perioperative outcomes in CKD patients is critical for optimizing preoperative risk stratification and postoperative management.

Serum albumin (SA) and serum creatinine (sCr) are two widely used biomarkers that reflect different aspects of renal and systemic health in CKD patients. Serum albumin, a marker for nutritional status and inflammation, is inversely linked to morbidity and mortality among CKD populations [10,11]. Low serum albumin levels are indicative of malnutrition, chronic inflammation, or proteinuria, all of which are common in CKD and linked to poor surgical outcomes [11,12]. On the other hand, serum creatinine, a byproduct of muscle metabolism, serves as an important marker of kidney function. High level of serum creatinine is a hallmark of reduced glomerular filtration rate (GFR), a defining characteristic of CKD [11]. However, the interpretation of serum creatinine alone can be confounded by factors such as muscle mass, age, and sex [13]. The serum albumin-to-creatinine ratio (sACR), a composite biomarker reflecting both renal function and systemic inflammation, has demonstrated superior prognostic utility across diverse clinical populations, including perioperative patients, outperforming isolated measurements of serum albumin (SA) or serum creatinine (sCr) in predicting adverse clinical outcomes [14–18]. Mechanistically, sACR integrates renal function (creatinine) and systemic inflammation/nutrition (albumin), both critical determinants of perioperative outcomes. However, to date, the prognostic role of sACR in perioperative CKD populations remains uncharacterized. To address this gap, we investigated the relationship between preoperative sACR and adverse clinical outcomes in surgical CKD patients, aiming to refine risk stratification frameworks and guiding targeted perioperative interventions.

## Method

### Data resource

This retrospective cohort study utilized the INSPIRE (Informative Surgical Patient dataset for Innovative Research Environment) database (Version: 1.3), a publicly available perioperative repository from Seoul National University Hospital (SNUH), South Korea, covering the period from 2011 to 2020. The dataset comprises 130,000 anonymized surgical cases, representing 50% of all eligible procedures. These cases were selected through systematic random sampling after applying exclusion criteria, including age <18 or >90 years, refusal to disclose admission data, or public media exposure of medical records. The dataset includes detailed information on operation anesthesia-related factors, diagnostic results, vital statistics, and laboratory data, as well as the prescription and administration of medications [19,20]. This study utilized de-identified data from the INSPIRE database, a publicly available research dataset. Dataset access permission was obtained by one of the authors (Bingwen Lin) under certification number 51729969. The original data collection study was approved by the Institutional Review Board (IRB) of Seoul National University Hospital (SNUH, IRB No. H-2210-078-1368), and informed consent was waived due to its retrospective nature. The Institutional Data Review Board (DRB) confirmed adequate de-identification of the data prior to public release (DRB No. BD-R-2022-11-02). As this study involved secondary analysis of publicly available, anonymized data, no additional ethical approval was required for the current analysis. The study followed the “Strengthening the Reporting of Observational Studies in Epidemiology” (STROBE) guidelines [21]. All procedures performed in this study were in accordance with the ethical standards of the Declaration of Helsinki.

### Study population

**Figure 1.**
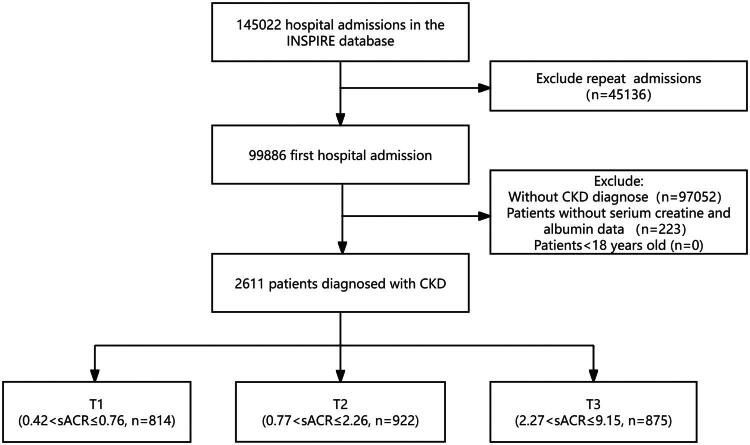
The flow chart of participants. INSPIRE, Informative Surgical Patient dataset for Innovative Research Environment; CKD, Chronic kidney disease.

Patients with CKD were retrospectively identified from the institutional database. CKD diagnoses were validated by clinicians using clinical practice guidelines and verified *via* ICD-10-CM codes (N18.1-N18.9). Patients without serum albumin and serum creatinine data and those under 18 years old were excluded. When considering instances of repeated hospitalizations, our analysis included only the first admission. Ultimately, the final analysis included a total of 2611 patients. The patient screening process is elaborately depicted in Figure 1.

### Data collection

Data were extracted from the INSPIRE database, encompassing comprehensive patient variables categorized into demographic, clinical, laboratory, and surgical parameters. Demographic details encompassed age, sex, body mass index (BMI), and the American Society of Anesthesiologists (ASA) score. Clinical and laboratory variables, collected within 24 h after hospital admission, comprised vital signs (heart rate, respiratory rate, blood pressure, temperature), blood tests (white blood cell count, platelet count, hemoglobin, albumin, creatinine, urea nitrogen, and electrolytes (sodium, potassium, calcium, and phosphorus), alanine aminotransferase (ALT), and aspartate aminotransferase (AST), and surgical metrics (emergency operation status, anesthesia type, surgery type, operating room time, operative time, anesthesia duration, and length of hospital stay). The baseline eGFR was calculated according to the Modification of Diet in Renal Disease (MDRD) Study Equation [22]. CKD stages 1–5 were based on eGFR: G1 (eGFR ≥90), G2 (60–89), G3 (30–59), G4 (15–29), and G5 (<15), all in ml/min/1.73m^2^. The sACR was determined at the time of admission by dividing serum albumin (g/dL) by serum creatinine (mg/dL). Patients were then stratified into three tertiles (T1: 0.42–0.76; T2: 0.77–2.26; T3: 2.27–9.15) based on their sACR values.

### Primary outcome

The primary outcome was postoperative in-hospital mortality.

### Statistical analysis

Continuous variables were summarized as mean ± standard deviation (normally distributed) or median with interquartile range (IQR) (non-normally distributed). Categorical variables were reported as frequency (percentage). For comparisons across the three sACR groups, continuous variables were analyzed using one-way ANOVA (for normally distributed data) or Kruskal-Wallis test (for non-normally distributed data). Categorical variables were compared using Pearson’s chi-square test. Given that the missing data percentages were all below 4% (Supplementary Table 1), we used simple imputation, replacing missing values with the mean for normally distributed variables and the median for skewed variables. To assess multicollinearity among the covariates, variance inflation factor (VIF) analysis was performed. A VIF threshold of 10 was used to identify variables with potential collinearity issues. As presented in Supplementary Table 2, both operating room time and duration of anesthesia had VIF values exceeding 10, suggesting the presence of multicollinearity between these two variables.

A univariate Cox regression analysis was conducted to determine the variables linked to in-hospital mortality. To quantify the relationship between sACR and in-hospital mortality, multivariable Cox proportional hazards models were applied to estimate hazard ratios (HRs) with 95% confidence intervals (CIs). Proportional hazards assumptions were assessed through the analysis of Schoenfeld residuals, and global tests for all covariates (including sACR groups) yielded non-significant p-values (*p* > 0.05 for all terms; Supplementary Figure 1). Four hierarchical models were developed to assess the independent relationship of sACR and primary outcome: Model 1: Unadjusted analysis. Model 2: Adjusted for sex, age, BMI, ASA score and CKD stage. Model 3: Additional adjustment for white blood cell count, hemoglobin, ALT, calcium, and phosphorus. Model 4: Further adjusted for hypertension, diabetes mellitus, emergency operation, surgery type and operative time.

The criteria for variable selection included: (1) Variables with a p-value < 0.05 in univariate analysis. (2) Clinically relevant confounders identified by our team’s expertise. (3) Variables that, upon inclusion, changed the regression coefficient of the base model by more than 10%, indicating their significant impact on the outcome. (4) Variables without multicollinearity, as indicated by a VIF value < 10.

sACR was analyzed both as a continuous variable and a categorical variable (tertiles: T1-T3).

Kaplan–Meier survival analysis was conducted, and the log-rank test was applied to assess significant differences in outcomes across the three groups stratified by sACR levels. To assess potential non-linear associations between sACR and in-hospital mortality, multivariable-adjusted restricted cubic splines (RCS) with four knots (at the 5th, 35th, 65th, and 95th percentiles) were utilized, adjusting for Model 4 variables. Additionally, a two-piecewise Cox regression model, also adjusted for Model 4 variables, was employed to determine the threshold effect of sACR on in-hospital mortality.

A series of sensitivity analyses was conducted to assess the robustness of the association between sACR and in-hospital mortality. First, a complete-case analysis was performed by excluding records with missing values. Second, multiple imputation using the mice package in R was applied to account for missing data and to validate the consistency of the results. Third, an E-value analysis was conducted to evaluate the potential impact of unmeasured confounding. Fourth, to avoid dialysis-induced creatinine variability, we excluded patients receiving hemodialysis or CRRT from the primary analysis and then reexamined the association between sACR and in-hospital mortality.

Subgroup analyses stratified by age, sex, emergency surgery status, ASA classification, eGFR classification, hypertension, and hemodialysis use were performed. Interaction effects were tested using likelihood ratio tests, with *p*-values for interaction reported to assess heterogeneity across subgroups. The analyses were conducted with R Statistical Software (Version 4.2.2, http://www.R-project.org, The R Foundation) and the Free Statistics analysis platform (Version 2.0, Beijing, China). A two-sided *P* value < 0.05 was considered statistically significant.

## Results

### Baseline characteristics

The study cohort comprised 2,611 patients with CKD undergoing surgery, stratified into tertiles based on preoperative sACR. The baseline characteristics of the study cohorts are demonstrated in Table 1. The average age was 61.1 ± 14.8 years, with 65.2% (*n* = 1,702) males. The median values for albumin, creatinine, and sACR were found to be 3.7 ± 0.6 g/dL, 3.2 ± 1.9 mg/dL, and 1.2(IQR: 0.7–2.6), respectively. The overall in-hospital mortality rate was 5.2%, 26.5% requiring ICU admission and a median hospital stay of 10 days (IQR: 5–18). Baseline characteristics revealed distinct gradients across groups: lower sACR tertiles (T1) were associated with younger age, advanced CKD stages, and higher proportions of emergency surgeries, while higher sACR tertiles (T3) had higher albumin and hemoglobin, lower creatinine and urea nitrogen), smaller deviations in electrolyte levels and shorter operating times and lower ASA scores. Postoperatively, higher sACR groups exhibited significantly reduced ICU admission rates, decreased CRRT utilization, and lower in-hospital mortality.

**Table 1.** Baseline characteristics of study participants by sACR tertiles.

Variables	Total (*n* = 2611)	sACR			*P* value
		T1 (0.42–0.76) (*n* = 814)	T2 (0.77–2.26) (*n* = 922)	T3(2.27–9.15) (*n* = 875)	
Demographics					
Age (years)	61.1 ± 14.8	54.6 ± 14.4	61.7 ± 15.1	66.4 ± 12.4	< 0.001
Sex, *n* (%)					0.221
Female	909 (34.8)	280 (34.4)	340 (36.9)	289 (33)	
Male	1702 (65.2)	534 (65.6)	582 (63.1)	586 (67)	
BMI (kg/m^2^)	23.4 (20.8, 26.0)	22.5 (20.8, 25.4)	22.5 (20.2, 25.7)	24.4 (22.4, 27.1)	< 0.001
CKD stage					< 0.001
1	56 (2.1)	0 (0)	0 (0)	56 (6.4)	
2	343 (13.1)	0 (0)	7 (0.8)	336 (38.4)	
3	645 (24.7)	0 (0)	197 (21.4)	448 (51.2)	
4	536 (20.5)	20 (2.5)	481 (52.2)	35 (4)	
5	1031 (39.5)	794 (97.5)	237 (25.7)	0 (0)	
Heart rate (bpm)	78.3 ± 15.3	79.2 ± 15.1	79.6 ± 15.9	76.3 ± 14.7	< 0.001
Respiratory rate (bpm)	17.7 ± 1.5	17.6 ± 1.4	17.7 ± 1.7	17.7 ± 1.4	0.373
NIBP_SBP (mmHg)	135.5 ± 20.3	139.8 ± 20.9	135.8 ± 20.7	131.1 ± 18.4	< 0.001
NIBP_DBP (mmHg)	77.9 ± 12.5	80.4 ± 12.9	77.0 ± 13.1	76.5 ± 11.2	< 0.001
Temperature (°C)	36.4 ± 0.4	36.5 ± 0.4	36.4 ± 0.5	36.3 ± 0.4	< 0.001
Comorbidities, *n* (%)					
Hypertension	623 (23.9)	138 (17)	231 (25.1)	254 (29)	< 0.001
Diabetes Mellitus	899 (34.4)	246 (30.2)	354 (38.4)	299 (34.2)	0.002
Heart failure	195 (7.5)	56 (6.9)	75 (8.1)	64 (7.3)	0.597
Laboratory tests					
White blood cell count (/nL)	7.2 ± 3.1	6.9 ± 3.0	7.5 ± 3.4	7.3 ± 2.9	0.002
Platelet count (/nL)	199.0 ± 73.3	187.1 ± 65.5	196.2 ± 78.6	213.0 ± 72.0	< 0.001
Hemoglobin (g/dL)	11.2 ± 1.9	10.4 ± 1.5	10.9 ± 1.7	12.4 ± 1.9	< 0.001
Albumin (g/dL)	3.7 ± 0.6	3.6 ± 0.5	3.6 ± 0.7	3.9 ± 0.5	< 0.001
Creatine (mg/dL)	3.2 ± 1.9	5.5 ± 0.4	3.2 ± 1.3	1.2 ± 0.3	< 0.001
sACR	1.2 (0.7, 2.6)	0.7 (0.6, 0.7)	1.2 (1.0, 1.5)	3.2 (2.6, 3.9)	< 0.001
Urea nitrogen (mg/dL)	36.0 ± 17.1	49.9 ± 13.2	37.6 ± 15.0	21.5 ± 9.2	< 0.001
eGFR, mL/min/1.73 m^2^	20.5 (10.7, 46.2)	10.4 (8.3, 10.9)	20.2 (15.0, 28.8)	54.8 (41.6, 69.9)	< 0.001
Potassium (mmol/L)	4.4 ± 0.6	4.6 ± 0.6	4.4 ± 0.6	4.3 ± 0.5	< 0.001
Sodium (mmol/L)	137.7 ± 3.5	136.9 ± 3.6	137.6 ± 3.6	138.5 ± 3.0	< 0.001
Calcium (mg/dL)	8.8 ± 0.7	8.8 ± 0.8	8.8 ± 0.7	8.9 ± 0.6	< 0.001
Phosphorus (mg/dL)	3.8 ± 0.9	4.4 ± 0.9	3.8 ± 1.0	3.4 ± 0.6	< 0.001
ALT (IU/L)	14.0 (11.0, 22.0)	13.0 (9.0, 20.0)	13.0 (9.0, 20.0)	17.0 (13.0, 27.0)	< 0.001
AST (IU/L)	20.0 (14.0, 26.0)	18.0 (13.0, 23.0)	19.0 (14.0, 26.0)	22.0 (18.0, 28.0)	< 0.001
Surgical conditions					
ASA score	2.7 ± 1.4	3.0 ± 1.6	2.9 ± 1.5	2.3 ± 0.9	< 0.001
Emergency operation, *n* (%)	455 (17.4)	207 (25.4)	197 (21.4)	51 (5.8)	< 0.001
Anesthesia type, *n* (%)					0.517
General	2111 (80.9)	653 (80.2)	754 (81.8)	704 (80.5)	
MAC	292 (11.2)	101 (12.4)	98 (10.6)	93 (10.6)	
Others	208 (8.0)	60 (7.4)	70 (7.6)	78 (8.9)	
Types of surgery, *n* (%)					< 0.001
Urological	831 (31.8)	344 (42.3)	301 (32.6)	186 (21.3)	
Gastrointestinal	380 (14.6)	102 (12.5)	131 (14.2)	147 (16.8)	
Cardiovascular	337 (12.9)	108 (13.3)	132 (14.3)	97 (11.1)	
Ophthalmic	253 (9.7)	55 (6.8)	89 (9.7)	109 (12.5)	
Orthopedic & Musculoskeletal	247 (9.5)	57 (7)	77 (8.4)	113 (12.9)	
Dermatologic	102 (3.9)	32 (3.9)	39 (4.2)	31 (3.5)	
Otolaryngologic	99 (3.8)	25 (3.1)	26 (2.8)	48 (5.5)	
Other	362 (13.9)	91 (11.2)	127 (13.8)	144 (16.5)	
Receiving hemodialysis, *n* (%)	309 (11.8)	131 (16.1)	131 (14.2)	47 (5.4)	< 0.001
Operating room time (hour)	2.8 (1.7, 5.1)	3.2 (1.8, 5.3)	3.1 (1.8, 5.3)	2.4 (1.4, 4.0)	< 0.001
Operative time (hour)	1.9 (0.9, 3.8)	2.2 (1.1, 4.0)	2.1 (1.0, 4.1)	1.6 (0.8, 3.0)	< 0.001
Duration of anesthesia (hour)	2.6 (1.4, 4.8)	2.9 (1.7, 5.0)	2.8 (1.5, 5.1)	2.2 (1.2, 3.8)	< 0.001
Length of hospital stay (day)	10.0 (5.0, 18.0)	14.0 (7.0, 19.0)	12.0 (6.0, 20.0)	7.0 (4.0, 13.0)	< 0.001
ICU admission, *n* (%)	693 (26.5)	234 (28.7)	294 (31.9)	165 (18.9)	< 0.001
In-hospital death, *n* (%)	135 (5.2)	49 (6)	60 (6.5)	26 (3)	0.001

Data are presented as Mean ± SD, Median (IQR) or *n* (%). *n* (%) indicates number (percentage).

*Abbreviations:* sACR: Serum albumin-to-creatinine ratio; BMI: Body Mass Index; NIBP_SBP: Noninvasive Blood Pressure Systolic; NIBP_DBP: Noninvasive Blood Pressure Diastolic; eGFR: estimated glomerular filtration rates; ASA score: American Society of Anesthesiologists score; MAC: Monitored Anesthesia Care; ALT: Alanine aminotransferase; AST: Aspartate aminotransferase.

### Univariate analysis for In-hospital mortality

The results of the univariate analysis revealed several significant associations with the primary outcome (Supplementary Table 3). Increased age, lower BMI, elevated white blood cell count, reduced platelet count, lower hemoglobin, decreased serum albumin, and elevated liver enzymes (ALT/AST) were identified as significant predictors. Additionally, MAC anesthesia had higher mortality risk compared to general anesthesia, while specific surgical types (e.g., gastrointestinal, ophthalmic, and orthopedic/musculoskeletal procedures), ICU admission, and receiving CRRT were independently associated with mortality.

### Association between sACR and in-hospital mortality

The Kaplan-Meier curve showed that patients in the T1 group (lowest sACR tertile) have the lowest survival probability compared to those in T2 and T3 groups. However, this difference did not reach statistical significance (*p* = 0.216, Supplementary Figure 2).

The multivariable Cox proportional hazards analysis of in-hospital mortality (total events = 135) found an inverse association between sACR and mortality risk (Table 2). In fully adjusted models, each unit increment in continuous sACR was independently associated with a 29% reduction in mortality risk (adjusted HR = 0.71, 95% CI: 0.61–0.83; *p* < 0.001) after controlling for demographic variables (sex, age, BMI, CKD stage), laboratory parameters (white blood cell count, hemoglobin, ALT, calcium, phosphorus), comorbidities (hypertension and diabetes mellitus), ASA score, emergency operation, surgery type and operative time. Hazard reductions were consistent in Model 2 (HR = 0.53, 95% CI: 0.45–0.63; *p* < 0.001) and Model 3 (HR = 0.57, 95% CI: 0.48–0.67; *p* < 0.001), though attenuated in the unadjusted model (HR = 0.87, 95% CI: 0.74–1.02; *p* = 0.088). Categorical analysis by sACR tertiles revealed a dose-response relationship, with the highest tertile (T3) exhibiting a 48% mortality risk reduction compared to lower levels (adjusted HR = 0.52, 95% CI: 0.33–0.82; *p* = 0.005). Trend analysis confirmed a significant monotonic decrease in mortality risk across ascending sACR tertiles (*P* for trend = 0.005, 0.015, and 0.002 for Models 2–4, respectively).

**Table 2.** Cox proportional-hazard ratios for In-hospital mortality across sACR tertiles.

Variable	Model 1		Model 2		Model 3		Model 4	
	HR (95% CI)	*P* value	HR (95%CI)	*P* value	HR (95% CI)	*P* value	HR (95%CI)	*P* value
sACR	0.87 (0.74–1.02)	0.088	0.53 (0.45–0.63)	< 0.001	0.57(0.48–0.67)	< 0.001	0.71 (0.61–0.83)	< 0.001
sACR, tertiles								
T1 (0.42–0.76)	1(Ref)		1(Ref)		1(Ref)		1(Ref)	
T2 (0.77–2.26)	0.95 (0.65–1.39)	0.795	0.65 (0.46–0.92)	0.016	0.65 (0.48–0.95)	0.013	0.56 (0.4–0.79)	0.001
T3 (2.27–9.15)	0.71 (0.44–1.17)	0.181	0.52 (0.33–0.81)	0.004	0.61 (0.4–0.98)	0.03	0.52 (0.33–0.82)	0.005
*P* for trend		0.21		0.005		0.015		0.002

Model 1: no adjusted.

Model 2: adjusted for sex, age, BMI, ASA score and CKD stage.

Model 3: adjusted for model 2 plus white blood cell count, hemoglobin, ALT, calcium and phosphorus.

Model 4: adjusted for model 3 plus hypertension, diabetes mellitus, emergency operation, surgery type and operative time.

*Abbreviations:* sACR, serum albumin-to-creatinine ratio; CKD, chronic kidney disease; HR, hazard ratio; CI, confidence interval.

### Restricted cubic spline and threshold effect analysis

**Figure 2.**
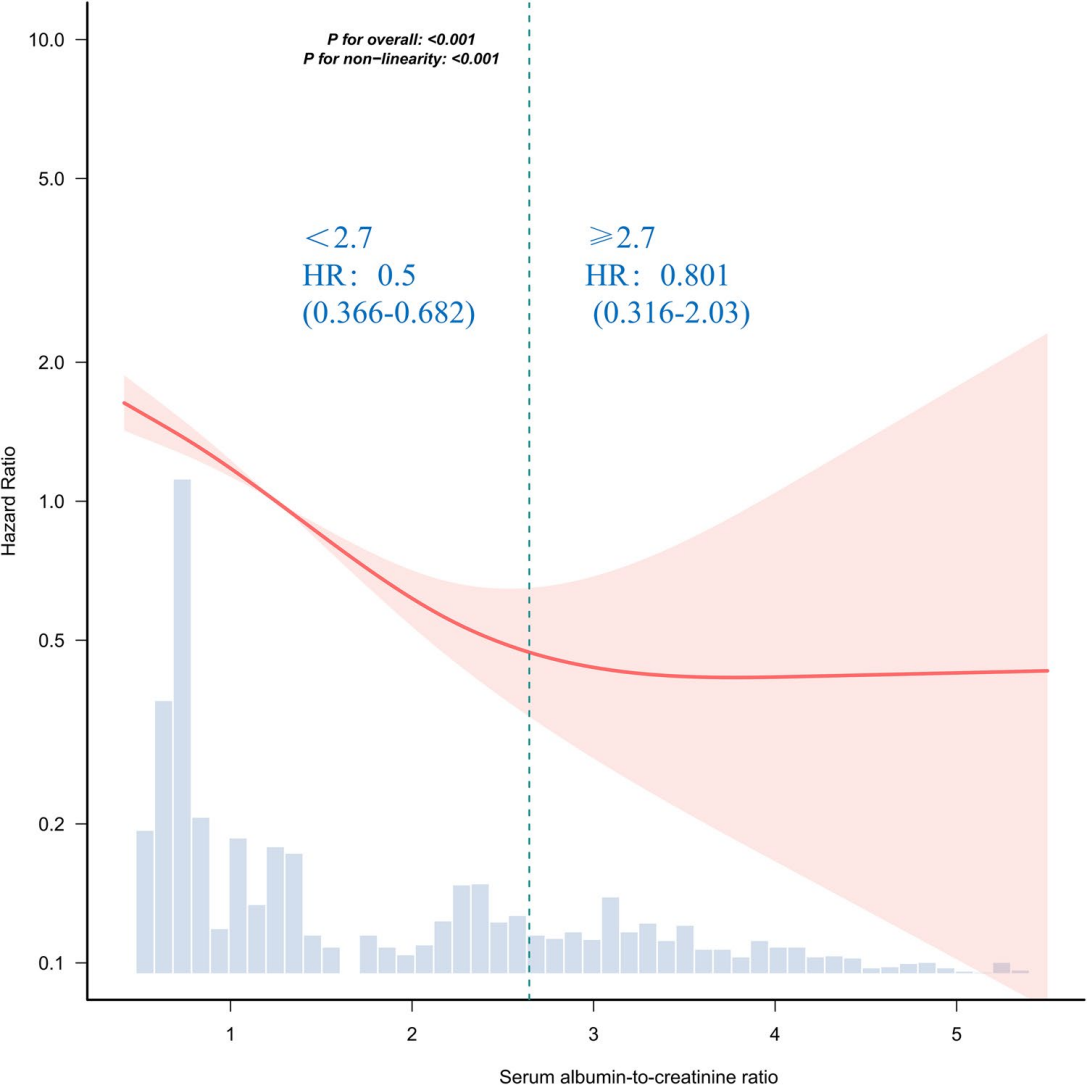
Association between serum sACR and in-hospital mortality. Solid and dashed lines represent the predicted value and 95% confidence intervals. Adjustment factors included sex, age, BMI, ASA score, CKD stage, white blood cell count, hemoglobin, ALT, calcium, phosphorus, hypertension, diabetes mellitus, emergency operation, surgery type, and operative time. Only 99% of the data is shown. sACR: serum albumin-to-creatinine ratio.

The restricted cubic spline analysis confirmed a non-linear (L-shaped) relationship between sACR and in-hospital mortality (*p* for non-linearity < 0.001; Figure 2). A threshold effect was identified at an sACR of 2.7, below which higher sACR levels were strongly associated with reduced mortality risk (HR: 0.5, 95% CI: 0.366–0.682, *p* < 0.001; Table 3). However, no significant association was observed for sACR ≥ 2.7 (HR: 0.801, 95% CI: 0.316–2.03, *p* = 0.6403).

**Table 3.** Threshold-effect analysis of the association between serum sACR and In-hospital mortality.

sACR	NO.	Adjusted model	
		HR (95% CI)	*P* value
<2.7	1994	0.5 (0.366–0.682)	< 0.001
≥2.7	591	0.801 (0.316–2.03)	0.6403
Likelihood Ratio test		–	0.042

*Note:* Adjusted for sex, age, BMI, ASA score, CKD stage, white blood cell count, hemoglobin, ALT, calcium, phosphorus, hypertension, diabetes mellitus, emergency operation, surgery type and operative time. Only 99% of the data is displayed.

*Abbreviations:* CI, confidence interval; sACR, serum albumin-to-creatinine ratio; HR, hazard ratio.

### Subgroup and sensitivity analysis

**Figure 3.**
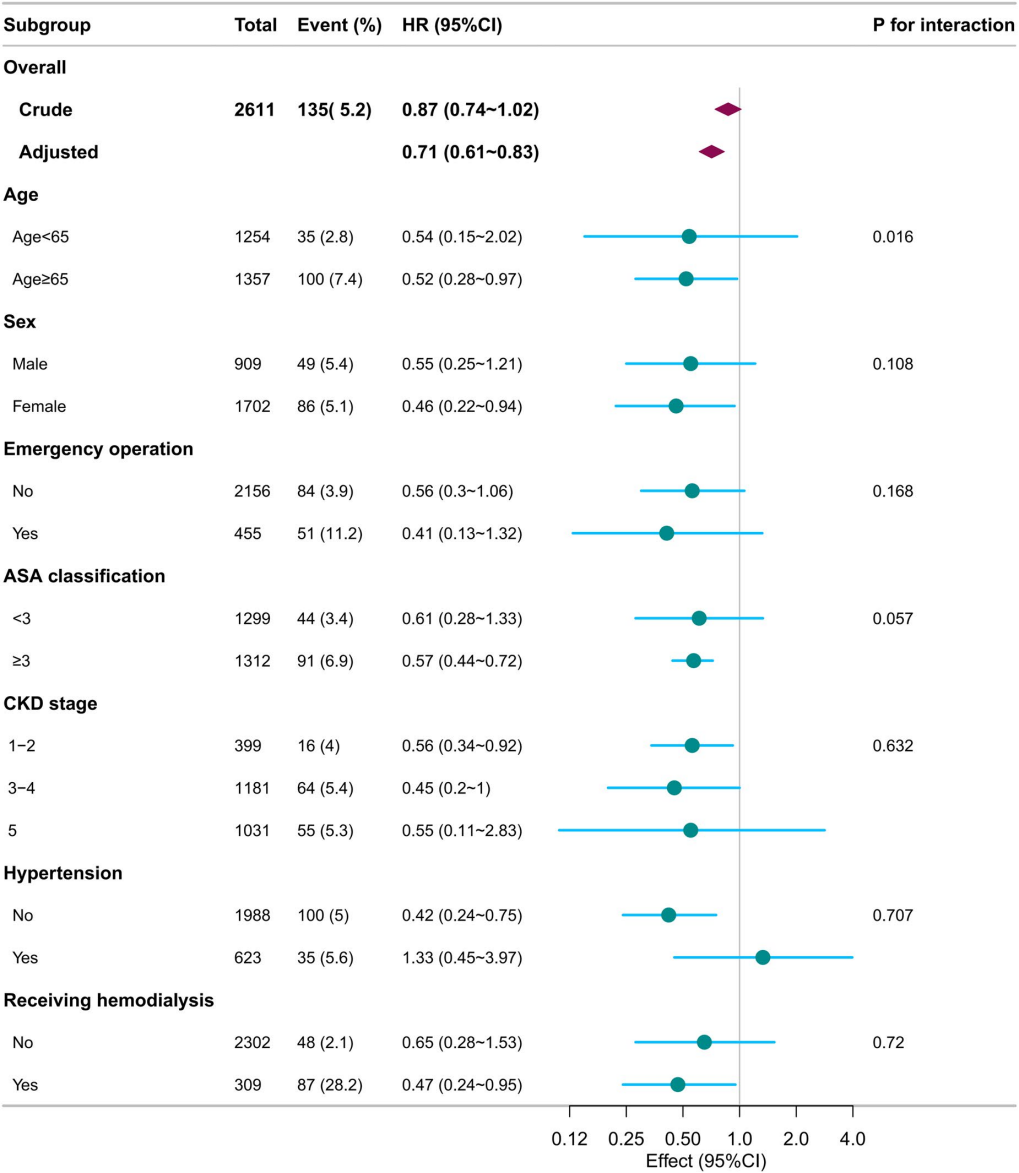
Subgroup analysis for the associations between serum sACR and in-hospital mortality. Each stratification adjusted for all confounders (Table 2, Model 4), except for the stratification factor itself. sACR: serum albumin-to-creatinine ratio.

Subgroup analyses revealed no statistically significant interactions (*p* > 0.05) for most variables, including sex, emergency operation, CKD stage, hypertension, and receiving hemodialysis. However, an exception was observed in the age subgroup analysis, where the protective effect of sACR on in-hospital mortality was more pronounced in patients aged ≥65 years compared to those <65 years (*P* for interaction = 0.016; Figure 3).

Finally, sensitivity analyses were conducted to assess the robustness of the observed association between sACR and in-hospital mortality. First, a complete-case analysis (excluding individuals with missing covariates) and multiple imputation (5 imputed datasets) were performed, both adjusted for the same covariates (Table 2, Model4). Results remained consistent across these methods (Supplementary Table 4 and Table 5). The third sensitivity analysis using E-values further evaluated potential unmeasured confounding (Supplementary Figure 3). The calculated E-value of 2.17 exceeded the relative risk (RR = 1.41) required for unmeasured confounders to explain away the observed association. Lastly, after excluding patients with dialysis or CRRT, the association between sACR and in-hospital mortality remained robust (adjusted HR 0.61, 95% CI: 0.45–0.84; *p* = 0.002; Supplementary Table 6), consistent with the primary analysis.

## Discussion

This study pioneers an exploration into the relationship between sACR and in-hospital mortality in perioperative patients with CKD. An L-shaped association was observed between sACR and in-hospital mortality, demonstrating a potential threshold effect identified at around 2.7. These findings indicate that sACR may serve as a significant biomarker to optimize risk assessment and management in CKD patients undergoing surgery.

Our results are consistent with those from prior investigations demonstrating the prognostic value of sACR in different disease settings [17,23–29]. For instance, in patients with severe acute pancreatitis, a lower sACR was associated with increased mortality and complications [17]. Similarly, in patients with non-ST-elevation acute coronary syndrome (NSTE-ACS), lower sACR levels were linked to higher all-cause mortality [18]. A retrospective study conducted by Karatas Met et al. found that low sACR was associated with increased in-hospital and long-term mortality and major stroke in patients undergoing carotid artery stenting [24]. Shen et al. [15] reported that an elevated preoperative ACR is linked to better overall survival among heart transplant recipients. Additionally, they found that higher ACR levels can forecast the likelihood of postoperative respiratory issues, renal dysfunction, liver injury, infections, and in-hospital mortality. Furthermore, research conducted by Hu Z et al. [25]demonstrated that low ACR levels are identified as independent risk factors for heightened in-hospital, ICU, and 30-day mortality rates among septic patients. However, prior to our investigation, no studies had explored the relationship between sACR and the risk of in-hospital mortality in CKD patients undergoing surgery. In contrast to previous studies, our research uncovered a negative yet nonlinear relationship between sACR and in-hospital mortality risk in perioperative CKD patients, characterized by a saturation effect. Specifically, sACR levels below the critical threshold of 2.7 demonstrated a stronger association with patient outcomes, whereas variations above this threshold did not significantly influence prognosis. This finding offers important insights into the dose-response relationship between sACR and in-hospital mortality, potentially aiding clinicians in more precise risk stratification. Moreover, our research specifically targeted CKD patients in the perioperative setting, a population with unique challenges and heightened risks. CKD patients undergoing surgery face additional perioperative stressors, such as anesthesia and potential nephrotoxic interventions, which can exacerbate underlying renal dysfunction. This contrasts with studies focusing on acute conditions like pancreatitis or sepsis, or chronic conditions like NSTE-ACS, where the primary clinical focus may differ [17,25,28,29]. This holistic approach provides a more nuanced understanding of the perioperative trajectory of CKD patients.

Serum albumin has long been recognized as a prognostic indicator in perioperative settings [30,31], though its interpretation is confounded by systemic inflammation, hepatic dysfunction, and volume overload [32,33]. Similarly, serum creatinine—central to renal assessment—is influenced by muscle mass, sarcopenia, and dietary protein intake, particularly in frail CKD populations [34–36]. The sACR integrates these complementary biomarkers to mitigate individual variability, providing a robust index reflecting inflammatory/nutritional balance (*via* albumin) and renal/metabolic status (*via* creatinine) [15]. Critically, its association with reduced mortality stems from four interconnected pathways: (1) Systemic inflammation and oxidative stress: Hypoalbuminemia (a negative acute-phase reactant) indicates chronic inflammation, driving NF-κB activation, IL-6/TNF-α elevation, endothelial dysfunction, and cardiovascular mortality [37,38], while oxidative stress exacerbates renal injury and proteinuria [39,40]; (2) Malnutrition-inflammation cascade: Low albumin signals protein-energy wasting, accelerating muscle catabolism (elevating creatinine) and immune impairment, compounded by inflammation suppressing albumin synthesis [41–43]; (3) Fluid dysregulation: Rising creatinine impairs sodium excretion, promoting volume overload and hypertension, while hypoalbuminemia reduces oncotic pressure, worsening myocardial strain and heart failure risk [44–46]; (4) Uremic toxin accumulation: Retention of protein-bound toxins (e.g., indoxyl sulfate) damages endothelial glycocalyx, induces insulin resistance, and synergizes with hypoalbuminemia to potentiate sepsis mortality [47–49]. The sACR demonstrates strong reproducibility, is readily derived from routine clinical data, and offers cost-effective perioperative risk stratification—enabling targeted interventions for surgeons, anesthesiologists, and nephrologists to optimize outcomes without additional testing.

This study, based on the INSPIRE database, is inherently limited by its retrospective cohort design. Firstly, as a single-center study, our findings may reflect local population characteristics and require multicenter validation for broader applicability.

Secondly, while our multivariable models (Models 2–4) and E-value analysis (≥2.1) support the robustness of the sACR-mortality association, the marked baseline imbalances—particularly the inverse relationship between sACR tertiles and CKD severity, necessitate cautious interpretation. These differences reflect the complex interplay between systemic inflammation (low albumin), renal dysfunction (high creatinine), and surgical risk. Future studies should employ propensity score matching to further isolate the prognostic role of sACR. Furthermore, due to inherent constraints in the INSPIRE database, residual confounding from unmeasured factors (e.g., frailty, nutritional status, or specific medications) cannot be fully ruled out. Although the observed association remains statistically robust, clinical interpretation must acknowledge this limitation. Future prospective studies incorporating comprehensive clinical data (e.g., frailty indices, detailed nutritional assessments, and medication records) are warranted to validate these findings and refine perioperative risk stratification. Thirdly, the inclusion of both high- and low-risk surgical procedures may attenuate the observed association, as mortality risk varies substantially across specialties. While we adjusted for surgical specialty, emergency status, and operative time, the absence of granular procedure-level data (e.g., specific surgical codes, complexity scores, or surgeon experience) precluded intra-specialty stratification. Consequently, residual heterogeneity in surgical complexity may persist within specialty groups, highlighting the need for future studies to incorporate detailed procedural metrics and validate findings in high-risk surgical subpopulations. Fourth, although VIF analysis identified collinearity, the multicollinearity between sACR and key variables (including creatinine, albumin and inflammatory markers) was not fully quantified. sACR’s clinical value lies in simultaneously integrating inflammatory (albumin) and renal (creatinine) dimensions—a composite advantage that single biomarkers cannot provide. Future work should quantify how much of the observed effect is uniquely attributable to this integration rather than to creatinine alone. Lastly, the observational design inherently precludes causal inference between sACR and clinical outcomes, allowing only for the description of associations. Therefore, prospective studies are warranted to further elucidate causal relationships and to corroborate our findings.

## Conclusion

In conclusion, this study found that lower preoperative sACR levels were associated with higher in-hospital mortality among surgical patients with CKD, with a potential nonlinear threshold at approximately 2.7. While the association appears robust, residual confounding from baseline differences (e.g., age, CKD stage), inclusion of both high- and low-risk surgeries (e.g. dermatology procedures), and moderate collinearity with kidney function variables may influence the observed relationship. Therefore, while sACR shows promise as a simple, integrative biomarker for perioperative risk stratification, its independent prognostic value should be interpreted cautiously. Future prospective studies with granular clinical data and refined surgical risk categorization are warranted to validate these findings and elucidate underlying mechanisms.

## Supplementary Material

Supplementary material.docx

## Data Availability

The datasets presented in this study are available in online repositories. Repository names and accession number(s) are provided below: https://physionet.org/content/inspire/1.3/
