# Supplementary material for: Association between the preoperative serum albumin-to-creatinine ratio (sACR) and in-hospital mortality in perioperative patients with chronic kidney disease: a cohort study based on INSPIRE database
Source: Ren Fail. 2026 Feb 4;48(1):2621525. doi: 10.1080/0886022X.2026.2621525 (PMC12875099; doi:10.1080/0886022X.2026.2621525)
Supplement: Supplementary material.docx [file IRNF_A_2621525_SM9868.docx]

**Association between the preoperative serum albumin-to-creatinine ratio (sACR) and in-hospital mortality in perioperative patients with chronic kidney disease：a cohort study based on INSPIRE database**

****Supplementary material****

Supplementary Table 1 Percentage of Missing Data for Each Variable in the Study Population

Supplementary Table 2 Variance Inflation Factor (VIF) of Covariates Included in the Multivariable Models

Supplementary Table 3 Univariate Analysis of Factors Associated with In-Hospital Mortality

Supplementary Table 4 Cox Proportional-Hazard Ratios for In-Hospital Mortality (Complete-Case Dataset)

Supplementary Table 5 Cox Proportional-Hazard Ratios for In-Hospital Mortality (Multiple-Imputation Dataset)

Supplementary Table 6 Cox Proportional-Hazard Ratios for In-Hospital Mortality Excluding Dialysis Patients

| **Supplementary Table 1. Percentage of Missing Data for Each Variable in the Study Population** | | |
| --- | --- | --- |
| Variable | Miss.frequency | Miss.percentage(%) |
| Demographics |  |  |
| Age | 0 | 0 |
| Sex | 0 | 0 |
| BMI | 36 | 1.3788 |
| CKD stage | 0 | 0 |
| Heart rate | 75 | 2.8725 |
| Respiratory rate | 77 | 2.9491 |
| NIBP_SBP | 77 | 2.9491 |
| NIBP_DBP | 77 | 2.9491 |
| Temperature | 77 | 2.9491 |
| **Comorbidities** |  |  |
| Hypertension | 0 | 0 |
| Diabetes Mellitus | 0 | 0 |
| Heart failure | 0 | 0 |
| **Laboratory tests** | 0 | 0 |
| White blood cell count | 44 | 1.6852 |
| Platelet count | 46 | 1.7618 |
| Hemoglobin | 25 | 0.9575 |
| Albumin | 0 | 0 |
| Creatine | 0 | 0 |
| sACR | 0 | 0 |
| Urea nitrogen | 19 | 0.7277 |
| eGFR | 0 | 0 |
| Potassium | 27 | 1.0341 |
| Sodium | 26 | 0.9958 |
| Calcium | 28 | 1.0724 |
| Phosphorus | 28 | 1.0724 |
| ALT | 23 | 0.8809 |
| AST | 23 | 0.8809 |
| **Surgical conditions** |  |  |
| ASA score | 97 | 3.7151 |
| Emergency operation | 0 | 0 |
| Anesthesia type | 0 | 0 |
| Types of surgery | 0 | 0 |
| Receiving dialysis | 0 | 0 |
| Operating room time (hour) | 0 | 0 |
| Operation time (hour) | 1 | 0.0383 |
| Duration of anesthesia (hour) | 7 | 0.2681 |
| Length of hospital stay (day) | 0 | 0 |
| ICU admission | 0 | 0 |
| In-hospital death | 0 | 0 |
| Abbreviations：sACR, Serum albumin-to-creatinine ratio; BMI, Body Mass Index; NIBP_SBP,Non-invasive Blood Pressure Systolic; NIBP_DBP, Non-invasive Blood Pressure Diastolic; eGFR, estimated glomerular filtration rates; ASA score, American Society of Anesthesiologists score; MAC, Monitored Anesthesia Care; ALT, Alanine aminotransferase; AST, Aspartate aminotransferase; | | |

| **Supplementary Table 2 Variance Inflation Factor (VIF) of Covariates Included in the Multivariable Models** | |
| --- | --- |
| Variables | VIF |
| Age | 1.246 |
| Sex | 1.261 |
| BMI | 1.126 |
| CKD stage | 1.687 |
| Heart rate | 1.267 |
| Respiratory rate | 1.216 |
| NIBP_SBP | 1.679 |
| NIBP_DBP | 1.65 |
| Temperature | 1.235 |
| Hypertension | 1.136 |
| Diabetes Mellitus | 1.18 |
| Heart failure | 1.195 |
| White blood cell count (/nL) | 1.425 |
| Platelet count (/nL) | 1.192 |
| Hemoglobin (g/dL) | 1.202 |
| Urea nitrogen (mg/dL) | 1.531 |
| eGFR, mL/min/1.73 m2 | 6.88 |
| Potassium (mmol/L) | 1.318 |
| Sodium (mmol/L) | 1.202 |
| Calcium (mg/dL) | 1.326 |
| Phosphorus (mg/dL) | 1.366 |
| ALT (IU/L) | 2.124 |
| AST (IU/L) | 2.306 |
| ASA score | 1.157 |
| Emergency operation | 1.254 |
| Anesthesia type | 1.403 |
| Types of surgery | 1.229 |
| Operating room time (hour) | **12.668** |
| Operative time (hour) | 9.397 |
| Duration of anesthesia (hour) | **14.347** |
| Receiving dialysis | 1.222 |
| ICU admission | 1.2 |
| Abbreviations：BMI, Body Mass Index; NIBP_SBP,Non-invasive Blood Pressure Systolic; NIBP_DBP, Non-invasive Blood Pressure Diastolic; eGFR, estimated glomerular filtration rates; ASA score, American Society of Anesthesiologists score; MAC, Monitored Anesthesia Care; ALT, Alanine aminotransferase; AST,Aspartate aminotransferase; | |

| ****Supplementary Table 3 Univariate Analysis of Factors Associated with In-Hospital Mortality**** | | |
| --- | --- | --- |
| Item | HR(95%CI) | ***P*** value |
| Age | 1.03 (1.02,1.05) | < 0.001 |
| Sex: Male vs Female | 0.86 (0.61,1.23) | 0.416 |
| BMI | 0.94 (0.91,0.98) | 0.006 |
| CKD stage |  |  |
| 1 | 1(Ref) |  |
| 2 | 0.53 (0.18,1.52) | 0.236 |
| 3 | 0.81 (0.31,2.09) | 0.658 |
| 4 | 0.97 (0.39,2.39) | 0.951 |
| 5 | 0.93 (0.38,2.28) | 0.872 |
| Heart rate | 1.0034 (0.9934,1.0135) | 0.51 |
| Respiratory rate | 0.96 (0.89,1.03) | 0.257 |
| NIBP_SBP | 0.9983 (0.9905,1.0062) | 0.676 |
| NIBP_DBP | 0.9916 (0.9787,1.0046) | 0.204 |
| Temperature | 1.37 (0.99,1.9) | 0.059 |
| Hypertension: Yes vs No | 1.35 (0.91,1.99) | 0.135 |
| Diabetes Mellitus: Yes vs No | 1.05 (0.74,1.49) | 0.784 |
| Heart failure: Yes vs No | 1.41 (0.89,2.23) | 0.142 |
| White blood cell count | 1.06 (1.02,1.1) | 0.004 |
| Platelet count | 0.9971 (0.9949,0.9992) | 0.008 |
| Hemoglobin | 0.87 (0.79,0.95) | 0.003 |
| Albumin | 0.54 (0.4,0.71) | < 0.001 |
| Creatinine | 0.9998 (0.9092,1.0995) | 0.997 |
| Urea nitrogen | 0.9998 (0.9092,1.0995) | 0.997 |
| eGFR | 0.9968 (0.9898,1.0039) | 0.383 |
| Potassium | 0.9 (0.69,1.16) | 0.42 |
| Sodium | 1.01 (0.97,1.06) | 0.518 |
| Calcium | 0.91 (0.72,1.16) | 0.451 |
| Phosphorus | 0.88 (0.74,1.05) | 0.162 |
| ALT | 1.0046 (1.0005,1.0087) | 0.027 |
| AST | 1.0083 (1.0042,1.0124) | < 0.001 |
| ASA score | 1.08 (0.99,1.19) | 0.098 |
| Emergency operation: Yes vs No | 1.27 (0.88,1.82) | 0.197 |
| Anesthesia type |  |  |
| General | 1(Ref) |  |
| MAC | 3.28 (1.63,6.62) | < 0.001 |
| Others | 0.75 (0.37,1.54) | 0.434 |
| Types of surgery |  |  |
| Urological | 1(Ref) |  |
| Gastrointestinal | 2.1 (1.14,3.85) | 0.017 |
| Cardiovascular | 1.69 (0.95,3.02) | 0.076 |
| Ophthalmic | 13.02 (5.29,32.03) | < 0.001 |
| Orthopedic & Musculoskeletal | 1.9 (1,3.6) | 0.049 |
| Dermatologic | 0.83 (0.32,2.11) | 0.691 |
| Otolaryngologic | 2.46 (0.83,7.31) | 0.104 |
| Other | 1.39 (0.74,2.62) | 0.307 |
| Operating room time (hour) | 0.96 (0.9,1.01) | 0.135 |
| Operation time (hour) | 0.94 (0.88,1.01) | 0.088 |
| Duration of anesthesia (hour) | 0.95 (0.89,1.01) | 0.088 |
| ICU admission: Yes vs No | 1.59 (1.1,2.3) | 0.014 |
| Receiving dialysis: Yes vs No | 4.49 (3.06,6.6) | < 0.001 |
| Data presentation: Continuous variables are presented as HR (95% CI), and categorical variables are compared using hazard ratios. Abbreviations:  BMI: Body Mass Index; NIBP_SBP: Non-invasive Blood Pressure Systolic; NIBP_DBP: Non-invasive Blood Pressure Diastolic; eGFR, estimated glomerular filtration rates; ASA score: American Society of Anesthesiologists score; MAC: Monitored Anesthesia Care; ALT: Alanine Aminotransferase; AST: Aspartate Aminotransferase; | | |

| **Supplementary Table 4 Cox Proportional-Hazard Ratios for In-Hospital Mortality (Complete-Case Dataset)** | | | | | | | | | | | |
| --- | --- | --- | --- | --- | --- | --- | --- | --- | --- | --- | --- |
| **Variable** | **Model 1** | |  | **Model 2** | |  | **Model 3** | |  | **Model 4** | |
|  | **HR (95%CI)** | ***P* value** |  | **HR (95%CI)** | ***P* value** |  | **HR (95%CI)** | ***P* value** |  | **HR (95%CI)** | ***P* value** |
| sACR | 0.87 (0.74–1.02) | 0.088 |  | 0.52(0.44–0.63) | < 0.001 |  | 0.58(0.49–0.7) | < 0.001 |  | 0.51 (0.43–0.61) | < 0.001 |
| sACR, tertiles |  |  |  |  |  |  |  |  |  |  |  |
| T1 (0.42-0.76) | 1(Ref) |  |  | 1(Ref) |  |  | 1(Ref) |  |  | 1(Ref) |  |
| T2 (0.77-2.26) | 0.95 (0.65–1.39) | 0.795 |  | 0.61 (0.42–0.9) | 0.012 |  | 0.62 (0.42–0.9) | 0.013 |  | 0.55 (0.38–0.81) | 0.002 |
| T3 (2.27-9.15) | 0.71 (0.44–1.17) | 0.181 |  | 0.42 (0.26–0.68) | 0.001 |  | 0.5 (0.31–0.82) | 0.006 |  | 0.46 (0.28–0.75) | 0.002 |
| P for trend |  | 0.21 |  |  | 0.001 |  |  | 0.004 |  |  | 0.001 |
| Model 1:no adjusted; | | | | | | | | | | | |
| Model 2: adjusted for sex, age , BMI, ASA score and CKD stage. | | | | | | | | | | | |
| Model 3: adjusted for model 2 plus white blood cell count, hemoglobin, ALT, calcium and phosphorus. | | | | | | | | | | | |
| Model 4: adjusted for model 3 plus hypertension, diabetes mellitus, emergency operation, surgery type and operative time. | | | | | | | | | | | |
| Abbreviations：sACR, serum albumin-to-creatinine ratio; CKD, chronic kidney disease; HR, hazard ratio; CI, confidence interval; | | | | | | | | | | | |

| **Supplementary Table 5 Cox Proportional-Hazard Ratios for In-Hospital Mortality (Multiple-Imputation Dataset)** | | | | | | | | | | | |
| --- | --- | --- | --- | --- | --- | --- | --- | --- | --- | --- | --- |
| **Variable** | **Model 1** | |  | **Model 2** | |  | **Model 3** | |  | **Model 4** | |
|  | **HR (95%CI)** | ***P* value** |  | **HR (95%CI)** | ***P* value** |  | **HR (95%CI)** | ***P* value** |  | **HR (95%CI)** | ***P* value** |
| sACR | 0.87 (0.74–1.02) | 0.088 |  | 0.52 (0.44–0.63) | <0.001 |  | 0.57(0.48–0.67) | < 0.001 |  | 0.71 (0.61–0.83) | < 0.001 |
| sACR, tertiles |  |  |  |  |  |  |  |  |  |  |  |
| T1 (0.42-0.76) | 1(Ref) |  |  | 1(Ref) |  |  | 1(Ref) |  |  | 1(Ref) |  |
| T2 (0.77-2.26) | 0.95 (0.65–1.39) | 0.795 |  | 0.67 (0.48–0.95) | 0.026 |  | 0.67 (0.48–0.95) | 0.027 |  | 0.6 (0.42–0.85) | 0.005 |
| T3 (2.27-9.15) | 0.71 (0.44–1.17) | 0.181 |  | 0.5 (0.31–0.78) | 0.003 |  | 0.56 (0.35–0.88) | 0.013 |  | 0.51 (0.32–0.82) | 0.006 |
| Model 1:no adjusted; | | | | | | | | | | | |
| Model 2: adjusted for sex, age , BMI, ASA score and CKD stage. | | | | | | | | | | | |
| Model 3: adjusted for model 2 plus white blood cell count, hemoglobin, ALT, calcium and phosphorus. | | | | | | | | | | | |
| Model 4: adjusted for model 3 plus hypertension, diabetes mellitus, emergency operation, surgery type and operative time. | | | | | | | | | | | |
| Abbreviations：sACR, serum albumin-to-creatinine ratio; CKD, chronic kidney disease; HR, hazard ratio; CI, confidence interval; | | | | | | | | | | | |

| **Supplementary Table 6 Cox Proportional-Hazard Ratios for In-Hospital Mortality Excluding Dialysis Patients** | | | | | | | | | | | |
| --- | --- | --- | --- | --- | --- | --- | --- | --- | --- | --- | --- |
| **Variable** | **Model 1** | |  | **Model 2** | |  | **Model 3** | |  | **Model 4** | |
|  | **HR (95%CI)** | ***P* value** |  | **HR (95%CI)** | ***P* value** |  | **HR (95%CI)** | ***P* value** |  | **HR (95%CI)** | ***P* value** |
| sACR | 0.92 (0.71–1.19) | 0.505 |  | 0.64 (0.47–0.87) | 0.005 |  | 0.67 (0.49–0.92) | 0.013 |  | 0.61 (0.45–0.84) | 0.002 |
| sACR, tertiles |  |  |  |  |  |  |  |  |  |  |  |
| T1 (0.42-0.76) | 1(Ref) |  |  | 1(Ref) |  |  | 1(Ref) |  |  | 1(Ref) |  |
| T2 (0.77-2.26) | 2.21 (1.1–4.45) | 0.026 |  | 1.13 (0.63–2.03) | 0.682 |  | 0.75 (0.42–1.36) | 0.347 |  | 0.73 (0.41–1.33) | 0.306 |
| T3 (2.27-9.15) | 0.98 (0.39–2.46) | 0.973 |  | 0.64 (0.3–1.38) | 0.254 |  | 0.48 (0.22–1.05) | 0.065 |  | 0.41 (0.19–0.89) | 0.024 |
| P for trend |  | 0.802 |  |  | 0.352 |  |  | 0.12 |  |  | 0.06 |
| Model 1:no adjusted; | | | | | | | | | | | |
| Model 2: adjusted for sex, age , BMI, ASA score and CKD stage. | | | | | | | | | | | |
| Model 3: adjusted for model 2 plus white blood cell count, hemoglobin, ALT, calcium and phosphorus. | | | | | | | | | | | |
| Model 4: adjusted for model 3 plus hypertension, diabetes mellitus, emergency operation, surgery type and operative time. | | | | | | | | | | | |
| Note: Use sample size= 2302; Abbreviations：sACR, serum albumin-to-creatinine ratio; CKD, chronic kidney disease; HR, hazard ratio; CI, confidence interval; | | | | | | | | | | | |
